# Management of Anemia in Lower-Risk Myelodysplastic Syndromes/Neoplasms With Novel Agents

**DOI:** 10.1200/OP-24-00910

**Published:** 2025-08-05

**Authors:** Fieke W. Hoff, Stephen S. Chung, Amer M. Zeidan, Yazan F. Madanat

**Affiliations:** ^1^ National Heart, Lung, and Blood Institute, National Institutes of Health, Bethesda, MD; ^2^ Department of Internal Medicine, Harold C. Simmons Comprehensive Cancer Center, UT Southwestern Medical Center, Dallas, TX; ^3^ Section of Medical Oncology and Hematology, Department of Internal Medicine, Yale Comprehensive Cancer Center and Yale University, New Haven, CT

## Abstract

Myelodysplastic syndromes (MDS) are a heterogeneous group of diseases that are prognostically stratified into lower-risk (LR-MDS) and higher-risk MDS on the basis of the International Prognostic Scoring System (eg, IPSS, revised IPSS, and molecular IPSS). Anemia is a hallmark of MDS and can lead to worsening of preexisting comorbidities, long-term RBC transfusion dependence, and profound fatigue. Although RBC transfusion support provides rapid relief of anemia-associated symptoms, it also carries a risk of iron overload and alloimmunization, and is associated with a decreased quality of life. Thus, many clinical trials and treatment strategies for LR-MDS focus on RBC transfusion independence (RBC-TI) as a primary end point. In this review, we discuss the updated treatment paradigm for anemia in LR-MDS. Novel insights in the pathogenesis of MDS and results from positive phase III clinical trials in LR-MDS have led to a growing number of therapeutic options (eg, luspatercept and imetelstat). Imetelstat was recently added as a new agent for patients who are refractory/resistant or ineligible for erythropoiesis-stimulating agent treatment on the basis of the randomized phase III IMerge trial, showing that imetelstat led to the primary end point of RBC-TI for ≥8 weeks in 40% of patients compared with 15% in patients receiving placebo. However, future clinical trials are needed to investigate the optimal sequencing of different agents and the potential of improving efficacy using combination of therapeutic strategies in LR-MDS.

## INTRODUCTION

Myelodysplastic syndromes/neoplasms (MDS) are characterized by clonal proliferation of the early hematopoietic progenitor cells, ineffective hematopoiesis, and variable degrees of peripheral blood cytopenias.^1^ Because of significant variability in clinical outcomes, prognostic scores are important to guide therapeutic decision making. The most used risk-stratification systems are the International Prognostic Scoring System (IPSS), the revised IPSS (IPSS-R), and the molecular IPSS (IPSS-M). An IPSS score of ≤1 and an IPSS-R score of ≤3.5 have generally been accepted as cutoff points to identify lower-risk MDS (LR-MDS).^2^ On the basis of the median overall survival (OS) of 4.6 years in patients with moderate-low IPSS-M, patients with an IPSS-M score of <0 have recently also been considered to have LR-MDS.^3^

Most patients with LR-MDS present with minor clinical symptoms and mild cytopenias at the time of diagnosis.^4^ In contrast to higher-risk MDS (HR-MDS), which carries an increased risk of progression to AML and is associated with a shortened OS, symptoms are mostly driven by anemia that is present in up to 85% of the cases.^5^ Symptomatic anemia can lead to worsening of preexisting comorbidities, long-term RBC transfusion dependence (RBC-TD), and profound fatigue affecting quality of life. Although RBC transfusion support provides rapid relief of anemia-associated symptoms such as fatigue, patients chronically receiving RBC transfusions are at increased risk of iron overload and alloimmunization, and RBC-TD has been associated with a decreased quality of life because of significant time investment, and increased mortality.^6-8^ Thus, many clinical trials and treatment strategies for LR-MDS focus on RBC transfusion independence (RBC-TI) as a primary end point.

## CURRENT TREATMENT STRATEGY FOR LR-MDS

Supportive care is a cornerstone in the management of all patients with MDS and consists of active disease surveillance, transfusion therapy, and management of other coexisting cytopenias (eg, platelet transfusions, RBC transfusions, and opportunistic infectious prophylaxis). Up to 90% of the MDS patients with anemia will require RBC transfusions at some point during their disease course, and approximately 40% of the patients with LR-MDS will become RBC transfusion–dependent.^9,10^ The first-line pharmacologic treatment for patients with an erythropoietin (EPO) level ≤200-500 U/L has historically been treatment with erythropoiesis-stimulating agents (ESAs). Overall response rates are approximately 20%-40% with a median duration of 15-18 months, and with particularly notable responses in patients with an endogenous EPO level <200 U/L.^11,12^ However, on the basis of data derived from the MEDALIST,^13^ COMMANDS,^14^ and IMerge^15^ phase III trials, treatment for LR-MDS has changed drastically.

**FIG 1.**
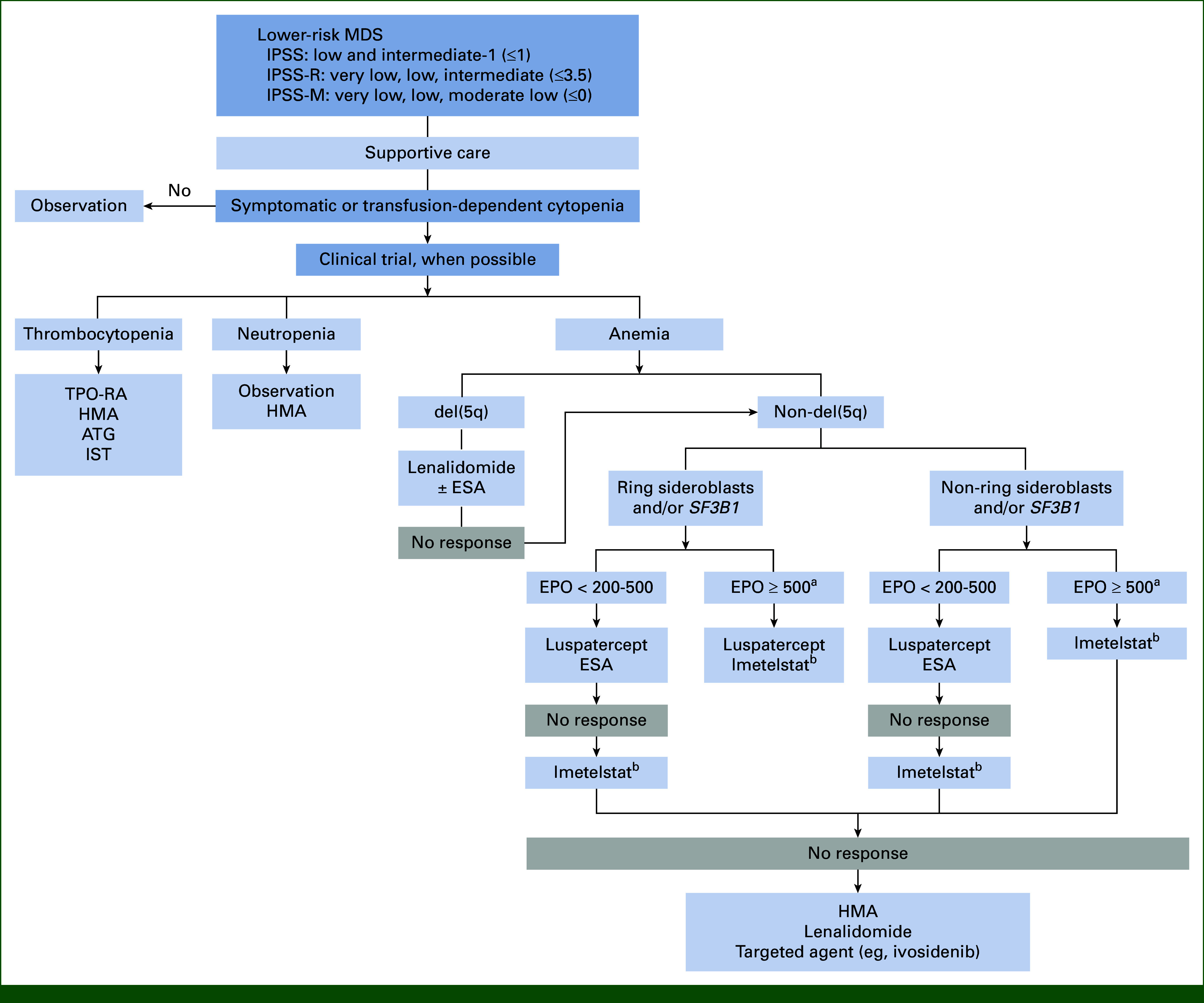
Treatment algorithm for lower-risk myelodysplastic syndrome (defined as IPSS score ≤1, IPSS-R ≤3.5, or IPSS-M ≤0). ^a^Or are ineligible to, have relapsed, or are refractory to ESA. ^b^Requires platelets > 50,000/μL and ANC > 1,000/μL. ANC, absolute neutrophil count; ATG, antithymocyte globulin; EPO, erythropoietin; ESA, erythropoiesis-stimulating agent; HMA, hypomethylating agent; IPSS, International Prognostic Scoring Systems; IPSS-M, molecular IPSS; IPSS-R, revised IPSS; IST, immunosuppressive therapy; MDS, myelodysplastic syndromes; TPO-RA, thrombopoietin receptor agonist.

If treatment with ESA fails or if patients are ineligible for ESA treatment because of a high baseline EPO level, treatment options may include lenalidomide, luspatercept, anti–T-cell immunosuppressive therapy (IST), hypomethylating agents (HMAs), or imetelstat, which recently approved by the US Food and Drug Administration (FDA) for patients with LR-MDS who are relapsed/refractory to or ineligible for ESA (Fig 1). In patients with deletion 5q (del(5q)), lenalidomide is the treatment of choice, on the basis of a response in approximately 70% of the patients with a median response duration of 2 years.^16^ If treatment no longer works, the treatment algorithm for patients with non-del(5q) should be followed. The MEDALIST, double-blind, phase III clinical trial investigated the use of luspatercept compared with placebo in LR-MDS patients with ring sideroblasts who were RBC-TD and relapsed/refractory to or ineligible for ESA therapy. Luspatercept acts as a ligand trap for transforming growth factor beta and inhibits downstream signaling of SMAD2/3, leading to improved RBC maturation. The study showed a 38% TI for ≥8 weeks during the first 1-24 weeks with luspatercept compared with 13% in the placebo group, which led to the initial approval of luspatercept for MDS with ring sideroblasts and/or *SF3B1* mutation for whom ESA therapy has failed. Despite improvement of RBC-TI with luspatercept, no improvement in health-related quality of life (HRQoL) was demonstrated. Subsequently, the COMMANDS phase III clinical trial compared luspatercept with epoetin alfa in RBC-TD, non-del(5q), LR-MDS patients (IPSS-R defined) with a baseline EPO level of <500 U/L who were ESA-naïve, resulting in approval of luspatercept in the frontline setting for treatment of LR-MDS. Patients had a median age of 74 years, a median transfusion burden of 3 units per 8 weeks in the 8 weeks immediately before the date of random assignment, and the majority had MDS with ring sideroblasts (73%) or mutated *SF3B1* (63%). Superiority with luspatercept was demonstrated on the basis of a 59% RBC-TI for at least 12 weeks with a concurrent mean hemoglobin increase of 1.5 g/L compared with 31% in the ESA group, and 48% a RBC-TI for 24 weeks compared with 29% in the ESA group. The median duration of 12-week RBC-TI was 127 weeks. Post hoc sub analyses showed 68% response rate for RBC-TI ≥24 weeks with luspatercept versus 39% with epoetin alfa for patients with ring sideroblasts, and 53% response rate with luspatercept versus 50% with epoetin alfa in patients without ring sideroblasts, suggesting that luspatercept could also be considered as first-line option for MDS without ring sideroblasts with a baseline EPO <500. A greater benefit was seen for patients with a low baseline transfusion burden (73%) compared with patients with a high baseline transfusion burden (47%). Luspatercept was associated with a shorter time to achieving a meaningful HRQoL improvement or delaying time to experiencing a meaningful HRQoL deterioration.^17^

HMAs (eg, azacitidine, decitabine, and decitabine-cedazuridine [oral decitabine]) are also FDA-approved for LR-MDS, although they have not led to an improvement in OS for those patients in randomized trials.^18,19^ No randomized phase III studies have shown superiority of either agent over the other.^20^ Results from the ASCERTAIN trial showed pharmacodynamic equivalence between oral decitabine-cedazuridine and intravenous decitabine.^21^ Ivosidenib, an IDH1 inhibitor, was recently FDA-approved for relapsed/refractory *IDH1*-mutated MDS (lower and higher-risk) on the basis of results from a single-arm phase I clinical trial showing complete remission (CR) + partial response rate of 38.9% and an objective response rate (CR + marrow CR) of 83.3%. Median treatment duration was 9.3 months with a median follow-up time of 27.1 months. Of note, at least four of the 19 patients had high or very high IPSS-R risk MDS at the time of diagnosis and six patients did not have an IPSS-R at the time of diagnosis due to being treated locally before trial enrollment.^22^ IST can be effective in subgroups of patients with LR-MDS, but it remains challenging to predict who are likely to respond and it is associated with toxicity, especially in older patients.^23,24^ Possible predictors include younger age, <5% bone marrow blasts, normal karyotype, presence of a paroxysmal nocturnal hemoglobinuria clone, short duration of TD, human leukocyte antigen-DR isotype positivity, or *STAT3*-mutant cytotoxic T-cell clones, with the most important predictor being hypocellular bone marrow. Finally, imetelstat is now FDA-approved in the United States and in the European Union as an additional agent for patients with LR-MDS who are refractory/resistant or ineligible for ESA treatment.

## MECHANISM OF ACTION OF IMETELSTAT

Imetelstat (RYTELO) is a first-in-class covalently lipidated 13-mer oligonucleotide that competitively binds with a high affinity to the RNA template region of human telomerase.^25,26^ This results in a direct and competitive inhibition of telomerase enzymatic activity and prevents telomere binding. In vitro and in vivo studies have showed that imetelstat inhibited telomerase activity in a dose-dependent manner, resulting in a loss of telomere length in hematologic malignancies including MDS.^27-29^ Inhibition of the telomerase also changed the cell cycle kinetics, causing delayed cell cycle progression through the G2 phase.

Telomerase is a ribonucleoprotein complex that contains the human telomerase reverse transcriptase (hTERT), and it is responsible for the synthesis and maintenance of telomeres.^30,31^ Telomeres correspond to the ends of eukaryotic chromosomes and are specialized structures containing unique TTAGGG repeats that protect the DNA from degradation, end-to-end fusion, rearrangement, and chromosome loss.^32,33^ In normal aging cells, the number of telomere repeats decreases after repeated cell divisions, and cell division stops at a certain telomere length, called the Hayflick limit.^34,35^

Shorter telomeres are frequently observed in the mononuclear cells of patients with MDS, and telomere dysfunction can be a critical factor driving MDS.^36,37^ Telomerase activity and hTERT expression, however, have been found to be often increased in MDS, which may drive the expansion of the malignant progenitor cell clone by circumventing cellular signaling, which would lead to replicative senescence or cell death.^38,39^ The combination of higher telomerase activity and hTERT expression, and shorter telomeres have been identified as poor prognostic factors in MDS, associated with higher progression to AML and a shorted OS.^40,41^ Given the importance of telomerase activity for cell survival, targeting telomerases may have an important therapeutic potential in MDS.^38^

**FIG 2.**
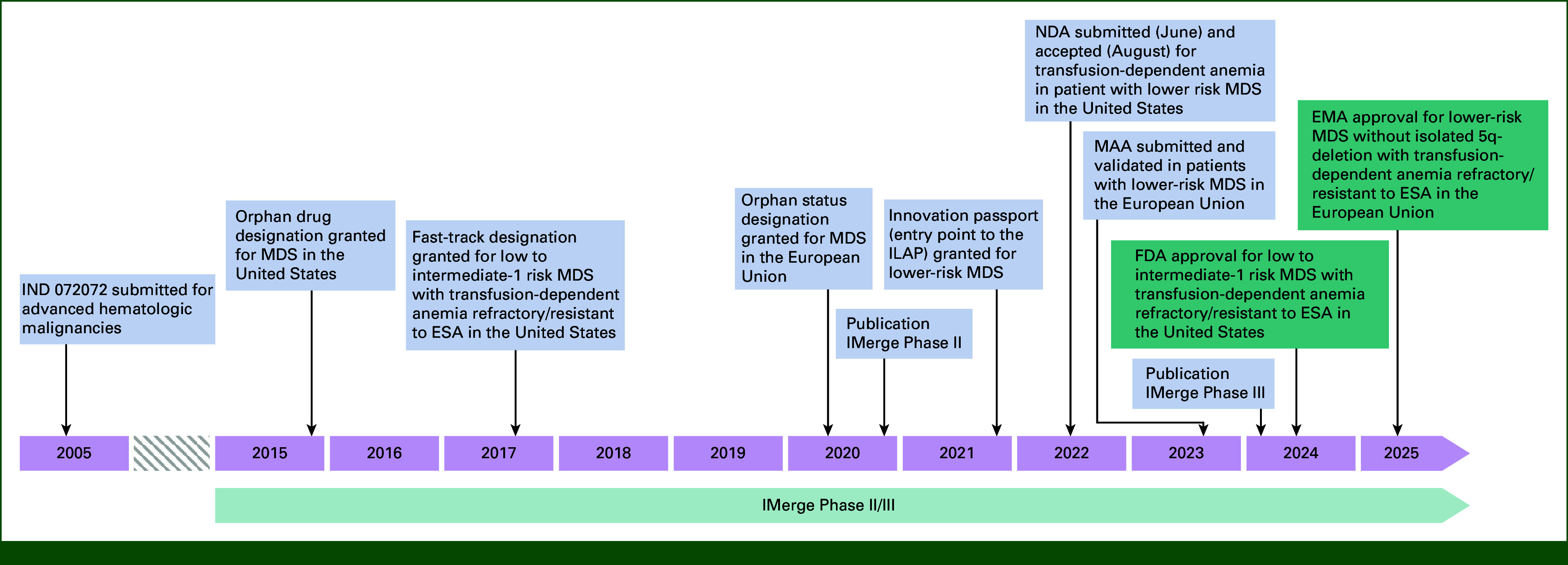
Timeline of the clinical development of imetelstat in myelodysplastic syndrome. ANC, absolute neutrophil count; EMA, European Medicines Agency; ESA, erythropoiesis-stimulating agent; EU, European Union; FDA, US Food and Drug Administration; ILAP, innovative licensing and access pathway; MAA, marketing authorization application; MDS, myelodysplastic syndromes; NDA, new drug application.

Figure 2 shows a timeline of the clinical development of imetelstat, including the FDA and European Medicines Agency (EMA) approvals. It is administered intravenously (7.1 mg/kg, which is equivalent to the imetelstat sodium 7.5 mg/kg used in the IMerge) in a single dose over 2 hours once every 4 weeks, and it is metabolized by nucleases to nucleotides of various lengths. It is primarily excreted by the kidneys and has not been shown to inhibit or induce CYP450 enzymes but is an inhibitor of OATP1B1 and OATP1B3.^25^

## RESULTS OF THE IMERGE CLINICAL TRIAL AND APPROVAL OF IMETELSTAT IN LR-MDS

The first dedicated clinical study for imetelstat in MDS was the phase II portion of the IMerge study in patients with LR-MDS, who had RBC-TD anemia requiring four or more RBC units over 8 weeks and who had MDS ineligible for or relapsed/refractory to ESA treatment.^42^ Fifty-seven patients were included with a median of 7 units per 8 weeks (range, 4-14) transfusion burden. Ninety percent of the patients had received previous ESA treatment, and 38 (67%) patients were HMA-naïve and lenalidomide-naïve. Patients received imetelstat sodium at a dose of 7.5 mg/kg (which is equivalent to the FDA label of the active compound of 7.1 mg/kg of imetelstat) once every 4 weeks and the median treatment duration was 8.2 months with a median of eight treatment cycles. The primary end point of the study was 8-week RBC-TI and was achieved in 37%, with 24-week RBC-TI in 23%. Median time to onset of 8-week TI was 8.3 weeks with a median duration of 65 weeks. A significant correlation between greater reduction of *SF3B1* variant allele frequency (VAF) and shorter onset time to achieve the longest RBC-TI was observed. Of note, 42% of 38 patients with non-del(5q) who were HMA-naïve and lenalidomide-naïve achieved the primary end point. Reversible neutropenia (67%) and thrombocytopenia (61%) were the most frequent treatment-emergent adverse events, mostly grade 3 or 4. Alanine transferase levels of any grade increased in 12% of the patients, with three (3%) patients developing grade 3 to 4.

On the basis of the results of the phase II portion showing durable achievement of RBC-TI, the IMerge phase III part was conducted comparing imetelstat versus placebo in RBC-TD, non-del(5q) LR-MDS patients who are ineligible, have relapsed, or are refractory to ESA.^15^ One-hundred and seventy-eight patients were randomly assigned 2:1 to receive imetelstat 7.5 mg/kg or placebo, administered as a 2-hour intravenous infusion once every 4 weeks. The median age was 72 and 73 years, 92% and 87% had received previous ESA, and 6% and 7% had previously used luspatercept, respectively. Patients who were previously treated with lenalidomide or HMA were excluded from the phase III main study. Both groups included 62% of the patients with ring sideroblasts. The median duration of treatment was 33.9 weeks on imetelstat and 28.3 week on placebo. The primary end point of RBC-TI for at least 8 weeks was reached in 40% of the patients in the imetelstat group versus 15% of the patients receiving placebo, and RBC-TI was sustained at 24-weeks in 28% compared with 3%. In patients receiving imetelstat, 8-week RBC-TI was reached in 45% of the patients with ring sideroblasts compared with 32% in patients without ring sideroblasts, versus 19% and 9% in patients receiving placebo with and without ring sideroblasts, respectively. Among imetelstat responders, the median hemoglobin increase at 8 weeks, 24 weeks, and 1 year was 3.6, 4.2, and 5.2 g/dL, respectively. Imetelstat was associated with a higher response rate compared with placebo in both patients with a baseline RBC transfusion burden of ≥4 to ≤6 units per 8 weeks (45% *v* 21%, *P* = .027) and in patients with a baseline RBC transfusion burden of >6 units per 8 weeks (34% *v* 7%, *P* = .023). The reduction in the VAF of *SF3B1*, *TET2*, *DNMT3A*, and *ASXL1* was greater with imetelstat than placebo. HRQoL was assessed during every clinical visits, including patient-reported fatigue using the functional assessment of chronic illness therapy (FACIT) fatigue scale.^43,44^ After two cycles, 50% of the patients treated with imetelstat sustained meaningful improvement in fatigue compared with 40% of the patients treated with placebo, and the mean change in FACIT-Fatigue score from baseline was consistently higher from cycle 3 onward. Data on OS or transformation to AML have not yet been reported. The key findings of the IMerge phase II/III are summarized in Table 1.

**TABLE 1.** Results of the Phase II/III Clinical Trials Evaluating Imetelstat Monotherapy in Adults With LR-MDS

Study	No.	Median Age, Years (range)	Imetelstat Dosing	8-Week RBC-TI, No. (%)	24-Week RBC-TI, No. (%)	1-Year RBC-TI, No. (%)	Median Duration 8-Week RBC-TI, Weeks
Steensma et al^42^ (phase II)	57	71 (46-83)	7.5 mg/kg^b^ once every 4 weeks	21 (37)	13 (23)	NA	65
Platzbecker et al^15^ (phase III)	118^a^	72 (65-75)	7.5 mg/kg^b^ once every 4 weeks	47 (40)	33 (28)	21 (18%)	51.6

Abbreviations: FDA, US Food and Drug Administration; LR-MDS, lower-risk MDS; MDS, myelodysplastic syndromes; NA, not assessed; RBC-TI, RBC transfusion independence.

^a^ Data refer only to patients randomly assigned to imetelstat arm.

^b^ 7.5 mg/kg imetelstat sodium that was evaluated in the IMerge phase II/III is equivalent to the active compound 7.1 mg/kg imetelstat that has received the FDA label for the treatment of LR-MDS.

Similar to the phase II study, the most common grade 3 or 4 adverse events in patients receiving imetelstat were neutropenia (68%) and thrombocytopenia (62%). The median duration of grade 3 or 4 neutropenia was 1.9 weeks, and 81% resolved to grade 2 or lower within 4 weeks. The median duration of grade 3 or 4 thrombocytopenia was 1.4 weeks, and 86% resolved to grade 2 or lower within 4 weeks. Severe neutropenia and thrombocytopenia were managed by dose reductions (67% and 47%, respectively) and delays (74% and 68%, respectively). Platelet transfusions were needed in 18% and 1.7% of patients treated with imetelstat and placebo, respectively, mostly used preventative for a platelet count <20,000/µL. Treatment discontinuation occurred in six (5%) and four (3%) patients, respectively.

## DISCUSSION

Management of LR-MDS with anemia is nuanced with a growing number of drugs, including the recent approval of imetelstat. Although imetelstat is approved for patients with LR-MDS who are RBC-TD and have relapsed or were refractory or ineligible to ESA, limited data are available regarding the optimal timing of initiation or dosing of imetelstat, as well as the subset of patients who will benefit most. On the basis of the first safety and efficacy analysis of the IMerge phase II trial showing that patients with non-del(5q) MDS who were naïve to treatment with a HMA and lenalidomide showed slightly higher response rates with an 8-week RBC-TI rate of 42% versus 37% in the overall population, patients were subsequently excluded for enrollment if they had a del(5q) cytogenetic abnormality or had received previous treatment with lenalidomide or HMA. Additionally, there are no robust data on the activity of imetelstat in patients after luspatercept failure or in patients who were treatment-naïve with a potential to receive ESA, as patients had to be previously ineligible to ESA, have relapsed, or were refractory to ESA to be trial-eligible.

However, pooled data analysis from the IMerge phase II, phase III, and the extension phase QTc study (Ventricular Repolarization substudy designed to further evaluate the long-term safety of imetelstat) investigated the effect of previous lines of therapy on the clinical activity of imetelstat. A total of 226 patients were included: 204 had previous treatment with ESA, 22 patients were ineligible for ESA, 35 had received previous luspatercept, 26 had received previous lenalidomide, and 22 were previously treated with a HMA. Although the numbers were small, all subsets experienced some clinical benefit from imetelstat treatment with ≥8-week and ≥24-week RBC-TI rates similar to rates reported in the IMerge.^45^ Of note, although the IMerge phase III excluded patients who had received previous treatment with lenalidomide or HMA, this has not been specified in the FDA/EMA label and patients could thus potentially receive imetelstat in these situations.

Furthermore, given different baseline patients characteristics between the phase III trials, with a RBC-TD of ≥4 units per 8 weeks in 37% of the patients compared with 100% of the patients in the COMMANDS and the IMerge, respectively, with patients enrolled on the COMMANDS trial being mostly ring sideroblast–positive (73%) and ESA-eligible, whereas all the patients in the IMerge trial were ESA-ineligible, have relapsed, or were refractory to ESA, and were either ring sideroblast–negative or ring sideroblast–positive, comparing the efficacy of luspatercept and imetelstat across trials is difficult, especially in the frontline setting for ESA-eligible patients.

The most common side effects of imetelstat were grade 3 to 4 neutropenia and thrombocytopenia, occurring in 68% and 62% of the patients, respectively. The majority of the cytopenias resolved within 4 weeks with dose delays and dose reductions, and this did not lead to an increased number of infection, febrile neutropenia, and bleeding events. Because of the risk of neutropenia and thrombocytopenia, the FDA label of imetelstat has clear recommendations for dose reductions for grade 3 and 4 adverse reactions, and requires a platelet count above 50,000/µL and an absolute neutrophil count (ANC) >1,000/µL. The degree of myelosuppression is clinically relevant, especially in the real world where patients have less access to platelet transfusions.

The FDA-approved dose for imetelstat is 7.1 mg/kg once every 4 weeks (which is equivalent to the 7.5 mg/kg dose used during the phase II), but there are limited data available supporting that this is the optimal dose to treat MDS. Phase I studies involving patients with solid and hematologic malignancies have provided information on drug pharmacokinetics and pharmacodynamics, demonstrating that 9.4 mg/kg once per week is the maximum dose associated with reversible myelosuppression constituting the dose-limiting toxic effect.^46^ Subsequently, a phase II study in essential thrombocythemia included 18 patients who received an initial dose of 7.5 mg/kg (n = 7) or 9.4 mg/kg (n = 11) of imetelstat IV once a week,^47^ and a pilot study in 33 patients with myelofibrosis including nine patients with MDS with ring sideroblasts, studying imetelstat 9.4 mg/kg once every 3 weeks or once every 4 weeks followed by once every 3 weeks,^48^ were performed. In this study, the most clinically significant side effect was myelosuppression, mandating dose reduction to 7.5 mg/kg or 6 mg/kg. On the basis of data from these nine patients, of whom three achieved RBC-TI for ≥8 weeks with imetelstat 7.5 mg/kg once every 4 weeks, 7.5 mg/kg was thus chosen as the dose for the imetelstat phase II/III studies in MDS.^49^ It is unclear whether a dose <7.5 mg/kg also has clinical efficacy in MDS, while reducing associated toxicity. Although dose modifications in patients who developed grade 3 or 4 adverse effects in the IMerge did not affect the RBC-TI rate, the mean dose intensity up to achievement of ≥8-week TI was 95.2% in responders and 90.5% in all patients.

HRQoL in patients with LR-MDS is mainly affected by symptoms related to cytopenia, time investment associated with RBC transfusions, treatment side effects, emotional well-being, and financial impact. Nevertheless, HR-QoL is often disregarded in clinical trials over surrogate end points such as RBC-TI. Although the MEDALIST trial failed to demonstrate significant benefit with regard to HRQoL, the IMerge resulted in sustained meaningful improvement in fatigue compared with placebo.

Although the question regarding the best timing of when to use imetelstat is still unanswered, the current national guidelines suggest sequencing imetelstat after luspatercept failure irrespective of *SF3B1* status or EPO level. For patients with high transfusion burden, however, where luspatercept responses are modest, and in patients with non-*SF3B1* MDS and a baseline EPO >500, who are ESA-ineligible and where luspatercept has not been studied, there may be a role for imetelstat favoring luspatercept in the frontline setting. Given imetelstat's high annual list price of 365,197 US dollars compared to the significantly lower costs of HMAs^50^ and supportive care, future research is needed to evaluate its clinical effectiveness from an economic perspective.

Finally, trials are currently ongoing exploring the potential role of treatment to prevent transfusion dependence in patients with LR-MDS to improve patient outcomes, and positive results from the Sintra-REV trial (ClinicalTrials.gov identifier: NCT01243476) have recently been published showing that low-dose lenalidomide (5 mg) in patients with LR-MDS with del(5q) improved hemoglobin levels and prolonged time to transfusion dependence from 11.6 months from beginning of treatment with placebo to 66.6 months with lenalidomide in non–transfusion-dependent patients.^51^ This randomized, double-blind, phase III trial was conducted in 22 sites and had a median follow-up time of 60.6 months. Patients had a baseline hemoglobin of 9.8 g/dL in both groups and all patients were ESA-naïve. Lenalidomide reduced the risk of transfusion dependency by 69.8%, and no increased probability of disease progression to AML or high-risk MDS was observed. The ELEMENT phase III trial (ClinicalTrials.gov identifier: NCT05949684) currently investigates the safety and efficacy of lupatercept versus epoetin alfa in reducing progression to RBC-TD in ESA-naïve, non–RBC-TD adult patients with anemia due to LR-MDS.^51^ Patients are eligible if they have a baseline EPO level <500 U/L and have clinically significant anemia defined as a score of moderate or worse on ≥1 Patient Global Impression of Severity item.

In conclusion, novel insights in the pathogenesis, improved risk stratification, and the approval of new agents on the basis of positive phase III clinical trials have improved the therapeutic landscape of MDS. Future trials are needed to investigate the optimal sequencing of different agents, dose optimization, and the potential of improving efficacy using combination therapeutical strategies in LR-MDS.
